# Transcytosis Assay for Transport of Glycosphingolipids across MDCK-II Cells

**DOI:** 10.21769/BioProtoc.3049

**Published:** 2018-10-20

**Authors:** Maria Daniela Garcia-Castillo, Wayne I. Lencer, Daniel J.-F. Chinnapen

**Affiliations:** 1 Division of Gastroenterology, Boston Children’s Hospital, Boston, Massachusetts 02115, USA; 2 Department of Pediatrics, Harvard Medical School, Boston, Massachusetts 02115, USA; 3 Department of Pediatrics, Harvard Digestive Diseases Center, Boston, Massachusetts 02115, USA

**Keywords:** Transcytosis, Endocytic sorting, Glycosphingolipids, Epithelial barriers, Drug delivery

## Abstract

Absorption and secretion of peptide and protein cargoes across single-cell thick mucosal and endothelial barriers occurs by active endocytic and vesicular trafficking that connects one side of the epithelial or endothelial cell (the lumen) with the other (the serosa or blood). Assays that assess this pathway must robustly control for non-specific and passive solute flux through weak or damaged intercellular junctions that seal the epithelial or endothelial cells together. Here we describe an *in vitro* cell culture Transwell assay for transcytosis of therapeutic peptides linked covalently to various species of the glycosphingolipid GM1. We recently used this assay to develop technology that harnesses endogenous mechanism of lipid sorting across epithelial cell barriers to enable oral delivery of peptide and protein therapeutics.

## [Background]

Transport of large molecules across the single-cell thick epithelial barriers lining mucosal surfaces and tight endothelial barriers lining vessels serving the heart muscle and brain occurs by an endocytic process that connects one side of these polarized cells with the other. This process is termed transcytosis (Garcia-Castillo *et al*., 2017). The absorption and secretion of immunoglobulins by receptor-mediated endocytosis and vesicular transport across the mucosa of the alimentary and respiratory tracts most famously typifies this process. Interest in transcytosis is also stimulated by the potential to harness this pathway for the delivery of therapeutic peptides and proteins across tight epithelial and endothelial barriers (Thuenauer *et al*., 2017).

Transcytosis is an active (ATP-driven) process. In some cases, transport of large molecules across tight epithelial and endothelial barriers can occur by passive diffusion around the cell through intercellular tight junctions (Fung *et al*., 2018). But aside from this small level of paracellular leakiness, physiologic meaningful paracellular transport of large molecules around cells occurs only when intercellular tight junctions are dismantled–usually under pathologic conditions. Non-specific and low levels of transcellular transport for large solutes may also occur by fluid-phase endocytosis and miss-sorting of cargo into transcytotic vesicles rather than into the late endosome/lysosome pathway, which receives the bulk of cargo internalized by non-receptor mediated mechanisms. Thus, any assay for transcytosis across healthy epithelial or endothelial barriers *in vitro* or *in vivo* needs to control for these confounding non-specific pathways.

**Figure 1.**
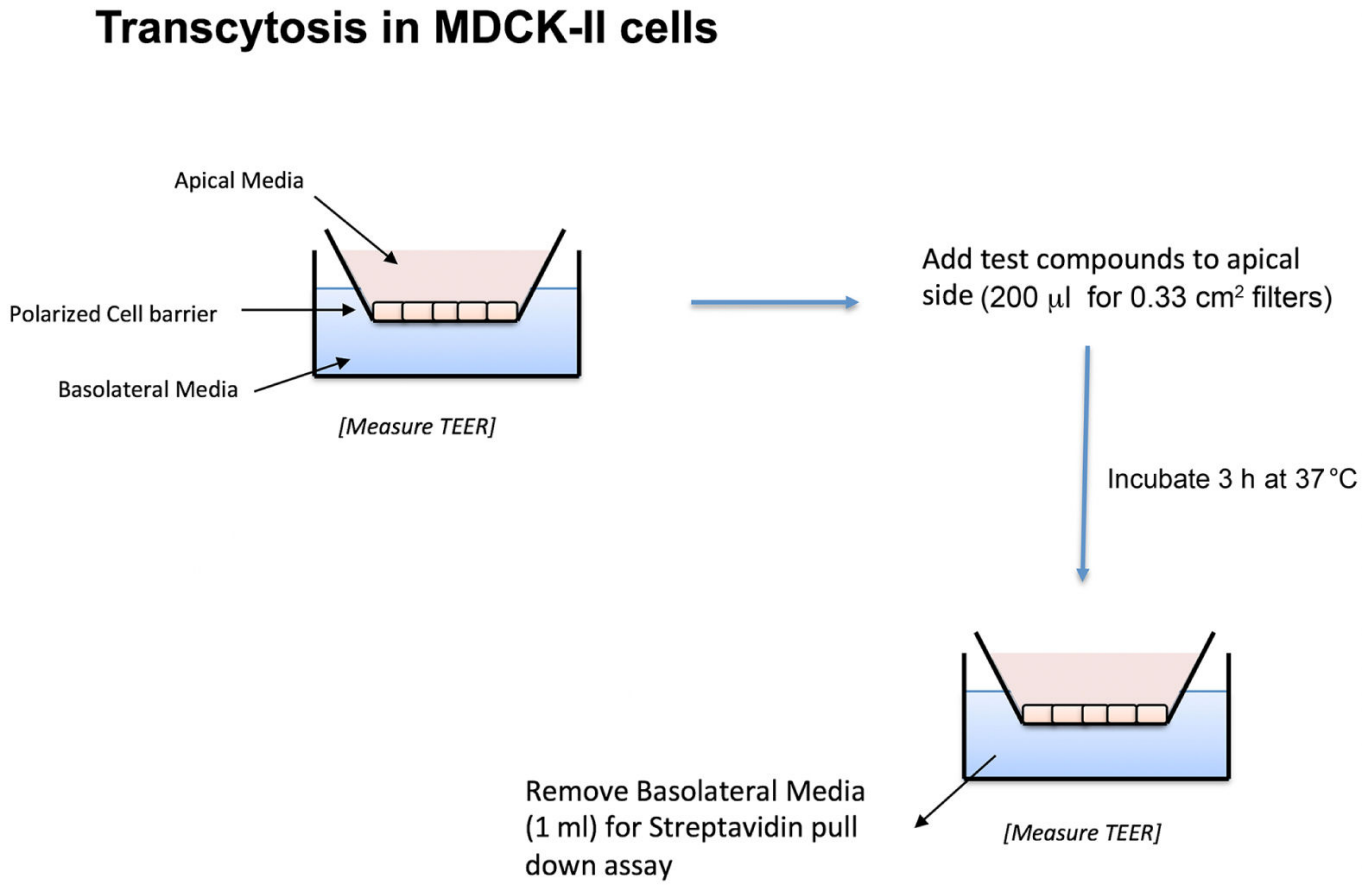
Schematic representation of MDCK-II transwell inserts and transcytosis assay

**Figure 2.**
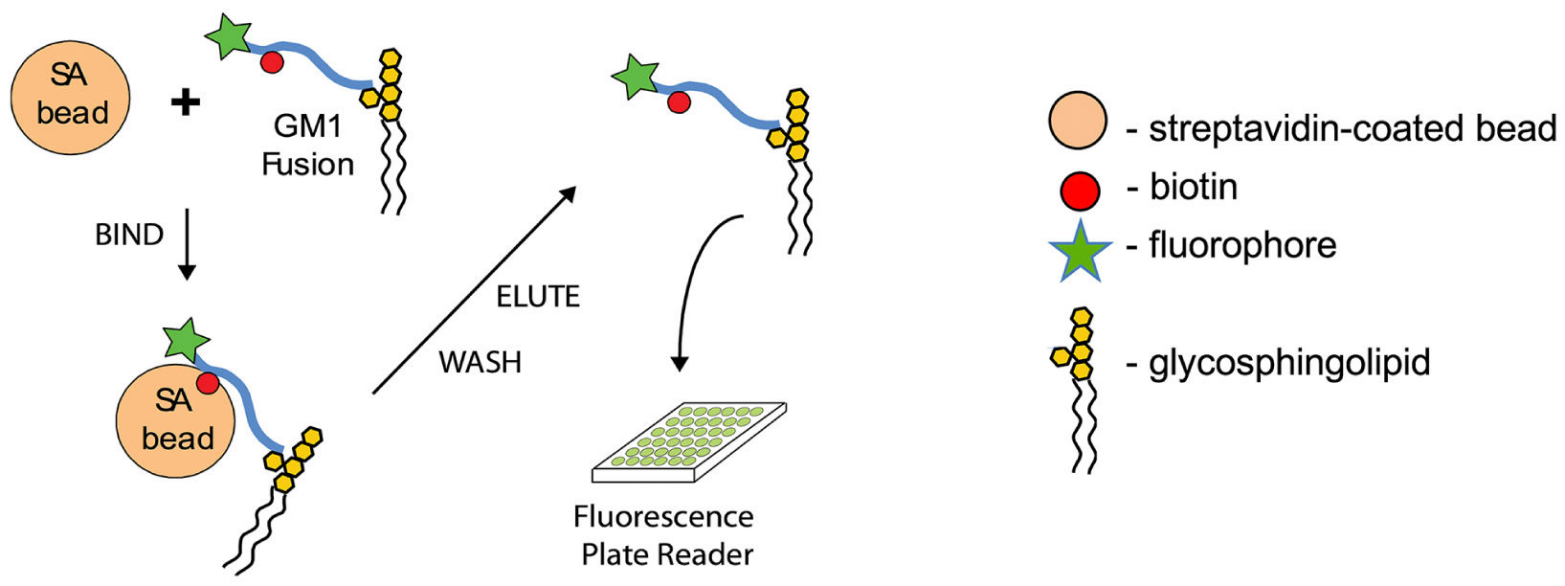
**Capture and read of biotinylated peptide–fusions from basolateral media** (Adapted from Figure 1A in Garcia-Castillo *et al*., 2018. Creative Commons Attribution License)

We have recently discovered that the endogenous sorting of glycosphingolipids across epithelial barriers can be harnessed for oral delivery of therapeutic peptides *in vivo* (Garcia-Castillo *et al*., 2018). Here we describe the *in vitro* assay using cultures polarized epithelial cells grown on transwell filters that we used to develop the technology that co-opts these endogenous mechanisms of lipid sorting for this purpose. The test compounds used to harness glycolipid sorting are modified to contain a biotin residue to allow for biochemical capture and a fluorophore for detection. Transwell inserts are composed of an inner chamber made with a permeable polycarbonate membrane support where the cells are seeded (Figure 1). After 3-7 days the epithelial cells assemble into a single cell thick monolayer with sealed tight junctions among cells. As such, the inner chamber of the transwell is exposed to the apical membrane of the epithelial monolayer and models the lumenal surface and can hold 200 μl of solution. The outer chamber–the chamber below the permeable support–is exposed to the basolateral surface of the monolayer and models the serosal surface of the epithelial barrier. This chamber holds 1 ml of basolateral solution. The assembly of the monolayer into a “tight” epithelial barrier with sealed functional tight junctions is routinely measured by passive resistance to small ion transport (TER–Transepithelial Resistance) measured by standard direct current electrophysiology or using the alternate current based machine EVOM. Test compounds are added to the apical chamber, and the assay is allowed to proceed to allow for transport across the barrier, where the basolateral chamber is then sampled for the transcytosed analyte using streptavidin-coated beads (Figure 2).

Mid-picomolar sensitivity can be achieved with this assay. Our basic approach can be used to measure transport by other molecules that enter the transcytotic pathway–such as for IgG that binds to the Fcγ-receptor FcRn that traffics in both directions across polarized epithelial monolayers (Nelms *et al*., 2017). The strength of our assay is the ability to directly, quantitatively, and sensitively measure the amount of compound transcytosed. Non-specific paracellular leak between cells versus real transport through cells is controlled in several ways, such as by using an analyte that cannot engage the transcytotic pathway (in our case the reporter peptide lacking fusion to the glycolipid carrier, or by fusion to a glycolipid with ceramide structure that cannot enter the transcytotic pathway), or by inhibiting endocytosis or transcytosis via a 4 °C temperature block, by chemical inhibition of endocytosis using Dyngo, and by siRNA knockdown of genes responsible for transcytosis as described in Garcia-Castillo *et al*. (2018).

## Materials and Reagents

**Figure 3.**
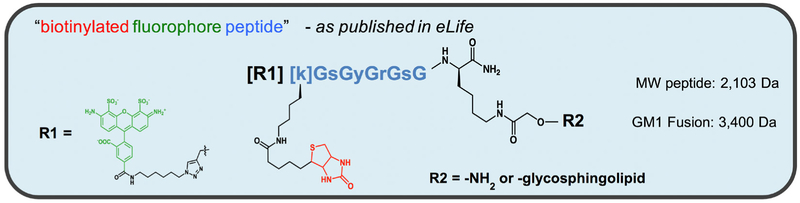
Structure of peptide-lipid fusion constructs used in transcytosis assay

Notes:

- Material and reagents are stored as per the manufacturer’s recommendation.
- Fluorescent peptides and glycosphingolipid-peptide fusions are used as described in Garcia-Castillo et al., 2018. The assay requires the presence of both a fluorophore and a biotin covalently attached to compound you want to measure (see Figure 3). Peptide fusions containing biotin and fluorophore attached to ganglioside GM1 were synthesized in-house using specialized methods described in (Garcia-Castillo et al., 2018). Precursor peptides were synthesized by New England Peptide (Gardner, MA, USA), and gangliosides were purchased from Dr. Sandro Sonnino (U. Milan, Italy). However, in theory, any macromolecule (peptide, protein, nucleic acid or chemical) containing both a fluorophore and biotin will work in this assay. An example compound is shown below. Here, lower-cased amino acids are depicting D-isomers. Since glycine (shown as upper-cased G) does not have chirality, the peptide essentially contains no L-amino acids that can be susceptible to degradation in vivo.

1. Pipette tips (USA Scientific, catalog numbers: 1126-7810, 1120-8810, 1121-3810)
2. 0.4 μm pore size Transwell® inserts (Corning, catalog number: 3413) *Note: Transwell® plates are kept at room temperature*.
3. 96-well Assay Plate (No Lid, Black Flat Bottom, Non-treated, polystyrene) (Corning, Costar®, catalog number: 3916)
4. Aluminium-foil (Fisher Scientific, FisherbrandTM, catalog number: S05356A)
5. MDCK-II cells (Parental line from American Type Culture Collection) (ATCC, catalog number: CCL-34) (Kind gift from Dr. Steven Claypool)
6. Fatty acid-free bovine serum albumin (Sigma-Aldrich, catalog number: A6003-10G) (lyophilized powder, essentially fatty acid free)
7. Pierce™ Streptavidin magnetic beads (Thermo Fisher Scientific, catalog number: 88817) *Note: Beads are stored at 4 °C per the manufacturer’s recommendation*.
8. Formamide (Sigma-Aldrich, catalog number: F9037-100ML)
9. Dimethylformamide (DMF) (Sigma-Aldrich, catalog number: 227056)
10. Biotin (Sigma-Aldrich, catalog number: B4501-1G)
11. DMEM 4.5 g/L D-glucose (Corning, catalog number: 10-013-CVR)
12. Pen/Strep, 100x (Thermo Fisher Scientific, Gibco™, catalog number: 15140148)
13. Fetal Bovine Serum (FBS) (Thermo Fisher Scientific, Gibco™, catalog number: A3160502)
14. Tris base (Sigma-Aldrich, catalog number: T1503-1KG)
15. NaCl (Sigma-Aldrich, catalog number: S9625-10KG)
16. Tween 20 (Sigma-Aldrich, catalog number: P2287–500ML)
17. 0.25% Trypsin-EDTA (Thermo Fisher Scientific, Gibco™, catalog number: 25200072)
18. PBS (Thermo Fisher Scientific, Gibco™)
19. EDTA (Sigma-Aldrich, catalog number: E4884-500G)
20. MDCK-II complete growth media (see Recipes)
21. Basolateral solution (see Recipes)
22. Apical solution (see Recipes)
23. TBS 10x (see Recipes)
24. 1x TBS-T (see Recipes)
25. 0.5 M EDTA pH 8.0 (see Recipes)
26. Elution buffer (see Recipes)

## Equipment

1. Portable Pipet-Aid® XP Pipette Controller (Drummond Scientific, model: 4-000-101)
2. Incubator Forma Scientific CO_2_ water-jacketed incubator (Thermo Fisher Scientific, model: Forma™ Series II, catalog number: 3110)
3. Centrifuge (Eppendorf, model: 5415C)
4. EVOM2 Epithelial Voltohmmeter (World Precision Instruments, model: EVOM2)
5. TECAN Spark multimode microplate reader (Tecan Trading, model: Spark®)
6. MagneSphere® Technology Magnetic Separation Stand (Promega, catalog number: Z5342)
7. Sonicator (Stainless Steel AquaSonic Ultrasonic cleaner, VWR, model: 50T)

## Software

1. GraphPad Prism 7
  GraphPad Software 7825 Fay Avenue, Suite 230 La Jolla, CA 92037 USA (https://www.graphpad.com/scientific-software/prism/)

## Procedure

### A. Day 1: Plating and polarizing cells

1. Trypsinization
  - Aspirate MDCK-II growth media from the stock cell culture flask used for passaging.
  - For a T-75 flask of MDCK-II cells, add 5-10 ml of warmed, sterile PBS. Gently tilt/rotate flask to wash the cells. Remove PBS.
  - For a T-75 flask, add 3 ml of 0.25% Trypsin-EDTA. Tilt/rotate flask to make sure cell surface is evenly coated.
  - Return to a 37 °C incubator with 5% CO_2_ under humidified conditions for 5-10 min.
  - Observe under a light microscope with phase contrast. If cells are properly detached, they will appear round and floating in suspension.
  - Collect cell suspension in conical tube and pipet cells 6-8 times up and down. No clumps should be observed. Cell suspension should be a homogeneous mixture.
2. Add MDCK-II cells at a density of 200,000 cells in 200 μl in complete growth media (see Recipes) to the apical side of a Transwell® insert (12-well 0.4 μm pore size Transwell®−65 mm). In addition, 1 ml complete growth media is added to the basolateral chamber.
  - Two Transwell® inserts are prepared for (Untreated) controls, 2 Transwell® inserts for reporter peptide, and 2 Transwell*®* per glycosphingolipid-peptide fusion tested.
  - Each condition is tested as biological duplicates (*i.e*., 2 Transwell® inserts per condition). Experiments must always include untreated and reporter-peptide controls.
3. Incubate the plate for 2-3 days to allow for polarization at 37 °C with 5% CO_2_ under humidified conditions.

### B. Day 3: Preparation of testing glycosphingolipid compounds

1. Centrifuge the tube containing lyophilized compound at 13,000 rpm (~13,800 × *g*) for 3 min to pellet the material to the bottom of tube.
2. Add 1 part volume of DMF (actual volume depends on stock concentration–for our compounds, this is ~60 μl), sonicate for 30–60 sec, followed by ~5 sec vortex.
  Notes:
  - Sonication is done in a bath sonicator (VWR, AquaSonic, model 50T).
  - You want to reach a high final concentration in the ~50-200 μM range (in a tube containing approximately 100 μg of lyophilized peptide [MW ~2,103], add 50 μl of DMF and 100 μl of water to obtain a stock solution in the ~50-200 μM range). The concentration of all test compounds is determined using NanoDrop (For the Alexa Flour 488 used in our studies, we use absorbance at 495 nm).
  - The solution may appear cloudy and orange-red at this point.
3. Add 2 parts H_2_O (for example 120 μl water to give a final volume of 180 μl) followed by a quick 5 sec vortex to reach a final 33% DMF in H_2_O.
  Notes:
  - The solution should be bright green and clear of particulates. If it is orange, then add either more DMF or H_2_O until it turns green. (The color change we observed is for an Alexa Flour 488 attached to the reporter peptide)
  - At this point, this stock solution will be used to make the final dilution into “Apical” media for adding onto cells.
4. To prepare this Apical test solution, dilute the appropriate volume of stock into Apical Solution (see Recipes) to reach a final concentration of 0.1 μM.
  Note: The goal is to have a final 1:1 ratio of lipid to dfBSA.

### C. Transcytosis in MDCKII cells

1. Check electrical resistance of MDCK-II Transwell® inserts after 2-3 days using EVOM Epithelial Voltohmmeter to measure integrity of tight junctions.
  Note: Acceptable electrical resistances of MDCK-II monolayers used for transcytosis experiments is in the 250Ω-300Ω range.
2. Prepare apical and basolateral solutions (see Recipes).
3. Wash transwells 2 times using serum-free DMEM. Replace media with apical (200 μl) and basolateral (1 ml) solutions in the respective chamber.
4. Allow cells to equilibrate for 15 min in a 37 °C/5% CO_2_ cell culture incubator.
5. Replace apical chamber with 200 μl apical solution containing 0.1 μM reporter peptide or 0.1 μM glycosphingolipid fusion.
  Note: Reporter peptide and glycosphingolipid fusion stocks range from 30 μM to 150 μM in 33% DMF/66% H_2_O.
6. Incubate for 3 h in a 37 °C/5% CO_2_ cell culture incubator.
7. To quantify transcytosed peptide and lipid-peptide fusions, collect basolateral media in pre-labeled Eppendorf tubes and proceed to streptavidin pull-down assay.
8. To calculate the apparent permeability coefficient (PAPP, cm/sec), apical chamber solution is also collected in pre-labeled Eppendorf tubes. A standard curve ranging from 0 nM to 200 nM is used to interpolate concentration of peptide or lipid-peptide fusion in apical chamber after 3 h.

### D. Streptavidin Pull-down Assay of basolateral media samples

1. Wash Streptavidin magnetic beads 3 times with TBS-T.
  Notes:
  - Beads are never vortexed. They are brought into solution in stock tube by gentle inverting.
  - Ten microliter beads are needed per sample.
    **Example:** For 12 samples, place 120 μl Streptavidin beads in an Eppendorf tube and wash 3 times each with 1 ml TBS-T using magnetic rack.
2. Resuspend 10 μl beads in 50 μl TBS then add to 1 ml basolateral sample
  **Example:** For 12 samples, resuspend 120 μl Streptavidin beads by adding 600 μl TBS. Then, add 50 μl Streptavidin beads to each basolateral sample.
3. Incubate basolateral samples with streptavidin beads overnight at 4 °C with head-over rotation covered in foil.

### E. Day 4: Elution and read-out of basolateral samples

1. Collect the beads using a magnetic rack and wash 3 times with TBS-T.
  Note: Each wash is done with 1 ml TBS-T and beads mixed by inverting.
2. Bound peptide or lipid-peptide fusions are eluted from beads by addition of 220 μl elution buffer (See Recipes) and boiling.
  Notes:
  - Invert the beads to ensure that they are in solution.
  - After beads are in solution, boil for 2 min at 65 °C in a heat block.
3. After boiling, collect the beads using a magnetic rack.
4. Pipet 100 μl of each sample × 2 (technical replicates) on a black 96-well plate.
5. Fluorescence is read on a TECAN Spark microplate reader for Alexa-488 channel and against a standard curve for each compound.
  - Settings:

**Table:** 

Mode	Fluorescence Top Reading
Excitation Wavelength	488 nm
Emission Wavelength	532 nm
Excitation Bandwidth	9 nm
Emission Bandwidth	20 nm
  - Sample standard curves (Figure 4)
  - A basolateral standard curve for each compound being tested is made in elution buffer ranging from 0 pM to 1,000 pM.
    - Make 1 μM solutions of peptide and lipid-peptide stocks in elution buffer.
    - Make 1 ml of 10 nM solutions (10 μl 1 μM + 990 μl elution buffer).
    - Make serial dilutions with elution buffer beginning with 1,000 pM:
      1,000 pM = 100 μl 10 nM + 900 μl elution buffer
      500 pM = 500 μl 1,000 pM + 500 μl elution buffer
      250 pM = 500 μl 500 pM + 500 μl elution buffer
      125 pM = 500 μl 250 pM + 500 μl elution buffer
      62.5 pM = 500 μl 125 pM + 500 μl elution buffer
      31.5 pM = 500 μl 62.5 pM + 500 μl elution buffer
      15.6 pM = 500 μl 31.5 pM + 500 μl elution buffer
      0 pM = elution buffer

### Data analysis

GraphPad Prism software is used to calculate the concentration of lipid-peptide fusion. As detailed above, a standard curve for each test compound is used to relate fluorescence intensity to a known concentration of peptide or lipid-peptide fusion. Absorbance at 495 nm is initially used to calculate stock concentrations. For detailed instructions on graphing and interpolating standard curves, the reader is referred to the GraphPad software website.

To calculate the apparent permeability coefficient (PAPP, cm/sec) a standard curve ranging from 0 nM to 200 nM is used to interpolate concentration of peptide or lipid-peptide solution remaining in the apical chamber after a 3 h continuous incubation.

The apparent permeability coefficient (PAPP, cm/sec) is calculated across cell monolayers grown in transwells based on the appearance rate of lipid-peptide fusions in the basolateral compartment over time:

Papp (cm/sec) = [VD/(A × MD)] × (DMR/Dt) Papp (cm/sec) = [cm^3^/(cm^2^ × mol)] × (mol/sec)

VD = apical (donor) volume (cm^3^) (*e.g*., 0.2 ml = 0.2 cm^3^)

MD = apical (donor) amount (mol)

A = membrane surface area (cm^2^) of apical (donor) chamber (*i.e*., transwell surface area = 0.33 cm^2^ in this protocol)

DMR/Dt = the amount of compound (mol) transferred to the basolateral (receiver) compartment over time (sec).

## Notes

1. This protocol is also used routinely in our lab with human intestinal T84 cells and human colonic Caco-2 cells.
2. Tight junction integrity must be confirmed using EVOM measurements.
3. To dissolve reporter peptide and glycosphingolipid-reporter peptide fusions first add DMF and sonicate for 30-60 sec, then add water.
4. Assay is done in serum-free DMEM.
5. The molar ratio 1:1 of compound to defatted-BSA is used.
6. When preparing compound solutions, keep solutions at 37 °C and never place diluted lipids on ice as they may form micelles.
7. A typical control for paracellular leak is a 4 °C temperature block to stop endocytosis/transcytosis machinery.
8. We use this assay in our lab to test the effect of gene silencing (using esiRNAs) and small molecule drugs (as is described for Dyngo-4a in Garcia-Castillo *et al*., 2018).
9. Using this assay, we performed structure-function studies of glycosphingolipid-peptide fusions containing modifications to the ceramide moiety. Also, using this assay, we tested for transcytosis of the incretin hormone GLP-1 (glucagon-like peptide 1).

## Recipes

1. MDCK-II complete growth media
  DMEM
  10% FBS
  1x Pen/Strep
  Store at 4°C
2. Basolateral solution (40 ml)
  40 ml DMEM
  400 mg defatted-BSA (df-BSA) (final concentration: 1% [w/v])
  Prepared fresh on the day of assay
3. Apical solution (40 ml)
  40 ml DMEM
  24.6 μl basolateral solution (Recipe 2, df-BSA final concentration: 0.1 μM)
  Prepared fresh on the day of assay
4. TBS 10x (1 L)
  24 g Tris base
  88 g NaCl
  Dissolve in 900 ml distilled water and pH 7.4
  Add distilled water to a final volume of 1 L
  Store at room temperature
5. 1x TBS-T (1 L)
  100 ml 10x TBS
  1 ml Tween 20
  900 ml distilled water
  Store at room temperature
6. 0.5 M EDTA pH 8.0 (1 L)
  186.1 g Na_2_EDTA to 800 ml MilliQ H_2_O
  Add NaOH to reach pH 8.0
  Add MilliQ H_2_O up to 1 L
7. Elution buffer (95% Formamide, 10 mM EDTA, 0.4 mg/ml biotin) (100 ml)
  95 ml 100% formamide
  2 ml 0.5 M EDTA, pH 8.0
  40 mg biotin
  3 ml MilliQ H_2_O

## Figures and Tables

**Figure 4.**
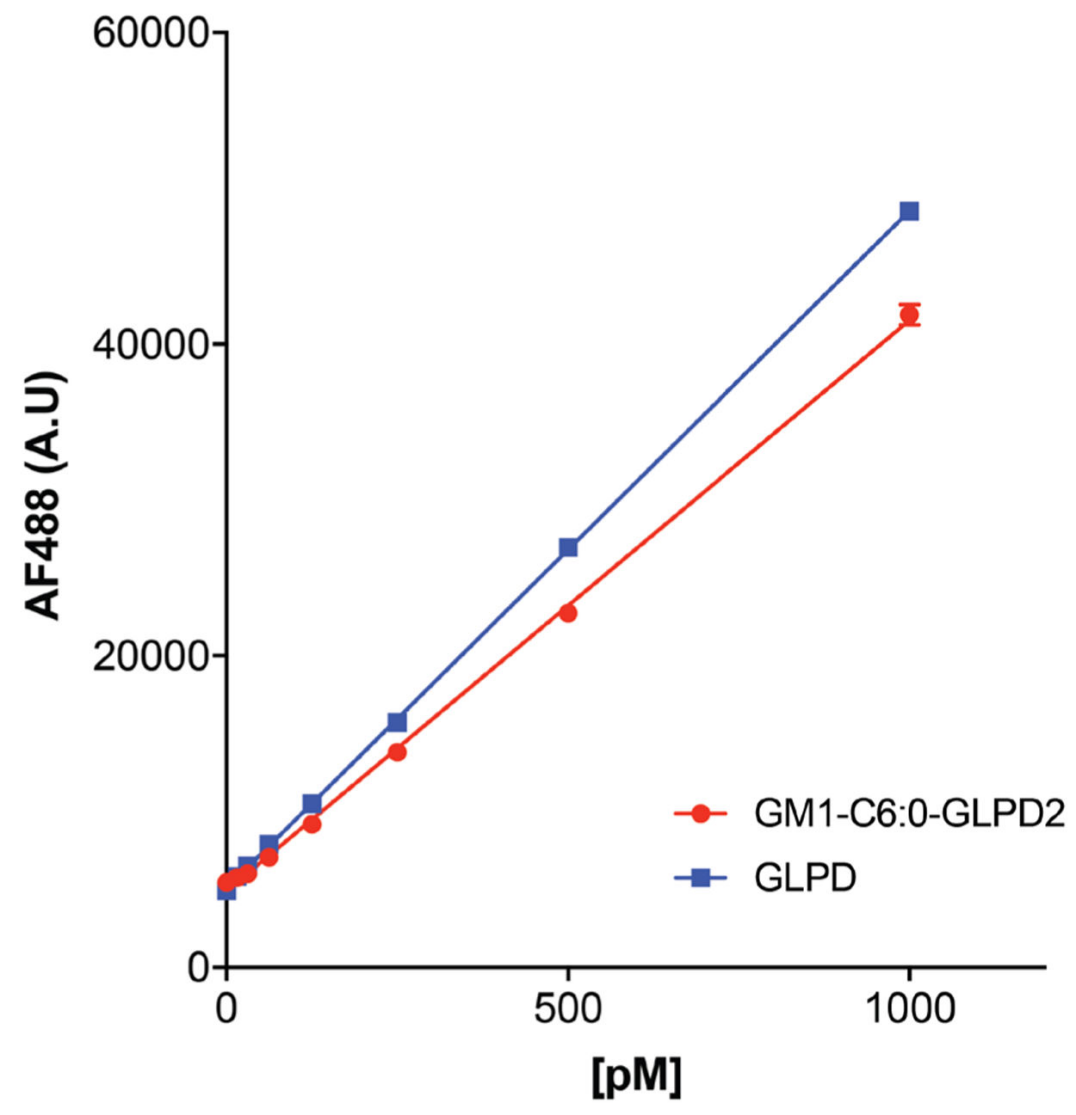
**Sample standard curves for peptide (blue) and lipid-peptide fusion (red) used to calculate the amount of transcytosed compound.** Plotted are the mean ± SEM of 3 technical replicates.
